# Development of invasive pink salmon ( *Oncorhynchus gorbuscha* Walbaum) eggs in a large Barents Sea river

**DOI:** 10.1111/jfb.15157

**Published:** 2022-07-23

**Authors:** Jaakko Erkinaro, Panu Orell, Jan‐Peter Pohjola, Mikko Kytökorpi, Henni Pulkkinen, Jorma Kuusela

**Affiliations:** ^1^ Natural Resources Institute Finland (Luke) Oulu Finland; ^2^ Natural Resources Institute Finland (Luke) Utsjoki Finland

**Keywords:** Barents Sea, egg development, hatching, invasive species, pink salmon, River Teno, spawning

## Abstract

The spawning and egg development of invasive pink salmon *Oncorhynchus gorbuscha* were investigated in the large River Teno in the Barents Sea area where they spawned for the first time on a large scale in 2021. The spawning period started in early August and egg development was rapid. All eggs were eyed by mid‐September and the first juveniles hatched in late September. In early October most eggs had hatched. Degree‐days in water temperature suggested that egg deposition had mostly taken place in early August. Early egg development is discussed in relation to possible consequences for survival.

After their introduction to the Russian White Sea basin in the late 1950s (Alekseev *et al*., 2019), pink salmon (*Oncorhynchus gorbuscha* Walbaum) gradually established naturally spawning populations in the White and Barents Sea Rivers, in both Russia and Norway (Sandlund *et al*., 2019). The current pink salmon population in the north‐east Atlantic area originates from a single introduction in 1985 from the Ola River, a North Okhotsk Sea drainage in the Magadan oblast, which has resulted in a self‐reproducing odd‐year population (Gordeeva *et al*., 2015) spanning over 18 successive generations until today.

The slow development turned into a rapid, widespread increase in the distribution and abundance of pink salmon in the North Atlantic area in 2017 (Sandlund *et al*. 2019; VKM *et al*., 2020). The high abundances persisted, especially in the Barents Sea area, in 2019 and even higher run sizes were detected in 2021 (VKM *et al*., 2020; Anon, 2021).

The life cycle of *O. gorbuscha* is widely studied in their native range in the Pacific area (*e.g*., Groot & Margolis, 1991; Quinn, 2018, and references therein), and many characteristics of the species' life history are well‐documented in the new Atlantic distribution area (*e.g*., Gordeeva *et al*., 2015; Alekseev *et al*., 2019). As timing of reproduction is one of the most critical adaptations of animal populations to their environment, the ideal spawning time of fish species or populations should result in emergence of juveniles at a date that optimizes their survival (Quinn, 2018). Although there are published studies revealing the development of *O. gorbuscha* eggs in different thermal regimes, to hatching and further until emergence, most of these studies have been carried out in hatcheries or controlled experimental facilities and little if any information is available from natural river habitats (Groot & Margolis, 1991, Quinn, 2018).

We observed spawning of *O. gorbuscha* in a large Barents Sea River, the subarctic River Teno (Tana in Norwegian) in the northern part of Finland and Norway (70°N) during and after an unpreceded *O. gorbuscha* run in this river in 2021 (Anon, 2021). Observations of spawning fish and their aggregations were made in several areas of the middle part of the Teno main stem by snorkelling and visual observations from the riverbank between the beginning of August and mid‐September.

Egg development and alevin hatching of *O. gorbuscha* were studied in three sampling areas located in the central part of the Teno mainstem, about 5 km downstream (Garnjarga, area no. 1), and 7 km (Kortsam, no. 2) and 19 km (Badda, no. 3) upstream from the confluence of the mainstem and the largest Finnish tributary, Utsjoki [see Erkinaro *et al*. (2019) for location]. Sampling took place approximately every second week between 7 September and 11 November. Three individual spawning redds were randomly selected and sampled in each of the three spawning areas during each sampling time. Sampling times and areas varied to some extent for logistical reasons and because of environmental conditions (Figure 2). Spawning redds were excavated by hand and shovel, and eggs/alevins were collected to a dip net held next to the redd downstream that effectively collected the eggs and alevins exposed to drift. We aimed at sampling 20–30 eggs/alevins per sampling period, but the actual catch varied between 7 and 115 (mean 33, s.d. 22) depending on the size of the redds and the environmental conditions. In addition, dead eggs were counted (Table 1).

**TABLE 1:** Number of sampled eggs (noneyed, eyed, dead) and alevins of *Oncorhynchus gorbuscha* in the River Teno between early September and early November (weeks 36–45) in 2021 (three sampling areas combined; see Figure 2)

Week	Noneyed	Eyed eggs	Dead eggs	Alevins	Total
36	30	204	10	0	244
37	0	126	1	0	127
39	0	60	1	2	63
40	1	137	22	299	459
43	0	1	0	94	95
45	0	3	0	117	120

Proportions of eggs and alevins were estimated using a Bayesian Dirichlet‐multinomial model. We assumed that the observed number of eggs/alevins sampled at spawning redd i at site s during week t that belong to life stages 1, 2 or 3 (noneyed, eyed, alevin) ($x_{i,1:3,s,t}$) follow a multinomial distribution:

(1) $$x_{i,1:3,s,t}\sim \text{Multi}\left(p_{1:3,s,t},N_{i,s,t}\right)$$

Here, $N_{i,s,t}$ is the total number of eggs/alevins sampled from spawning redd i at site s during week t and $p_{1:3,s,t}$ is the vector of probabilities that an individual sampled at site s during week t belongs to life stages 1, 2 or 3. Furthermore, an uninformative Dirichlet‐prior distribution was given for parameter $p_{1:3,s,t}$ as:

(2) $$p_{1:3,s,t}\sim \text{Dirich}\left(1,1,1\right)$$

Note that only individuals sampled at the same site during the same week share a common prior distribution, that is, the individuals in different site/week combinations are considered independent from each other. We chose this approach because of simplicity, but a hierarchical structure could have been used, for example across the sites in the same week. This would have likely pushed the estimated proportions closer to zero in cases where zero individuals of a certain category were observed at multiple sites (see Table 1).

Water temperature in the main stem of the Teno River (Figure 1) was measured by an automatic logger station in the lower part of the main stem (NVE; station Id 234.18.0, latitude 70.07034 N, longitude 28.01601 E, https://hydapi.nve.no).

**FIGURE 1:**
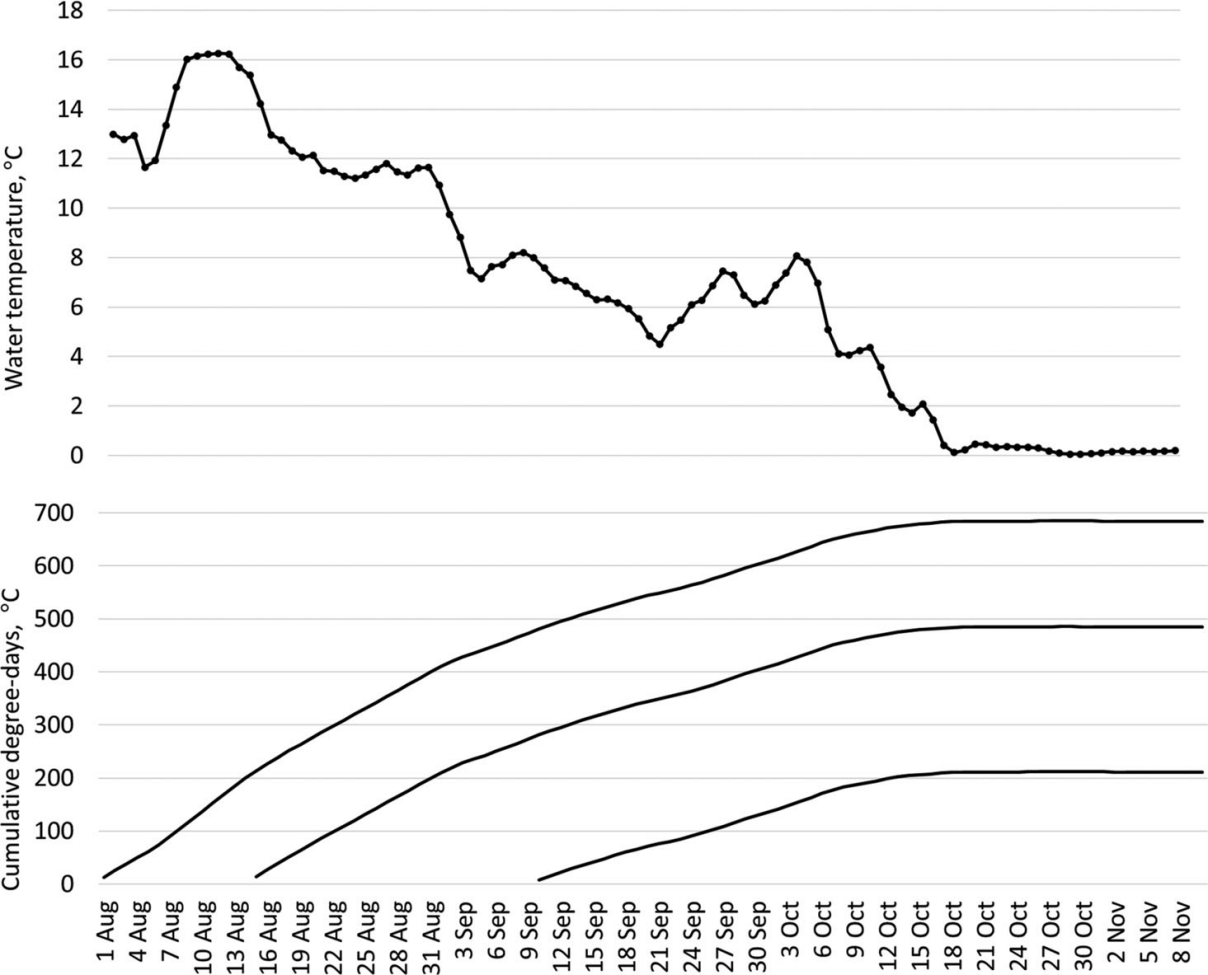
Mean daily water temperatures (°C) between 1 August and 11 November (upper panel), and three different cumulative degree‐day (°C) scenarios (lower panel) starting from 1 August, 15 August and 10 September, all within the observed *Oncorhynchus gorbuscha* spawning period in the Teno River in 2021

The main aggregations of spawning *O. gorbuscha* were mostly located in areas very close to the shoreline (*c*. 1–30 m) although the channel width was about 200 m in the monitored stretches of the Teno River. Most redds were located in shallow water (30–60 cm) with gravel‐cobble substrate and relatively swift flow velocities (*c*. 0,3–1 m/s). These characteristics were in good agreement with earlier published information on the spawning site requirements of *O. gorbuscha* (Groot & Margolis, 1991, Quinn, 2018; Alekseev *et al*., 2019).

In all three sampling areas some noneyed eggs (2%–37%, estimated mean proportions using a Bayesian Dirichlet‐multinomial model; Figure 2) were still present in early September (week 36), whereas in mid‐September (week 37), all remaining eggs were already eyed and some hatched juveniles were found in late September (week 39; Table 1 and Figure 1). In early October (week 40) a mix of eyed eggs (23%–45%) and hatched alevins (55%–75%) were detected in redds, and in late October almost only alevins were found (82%–97%). In November (week 45), river ice formation allowed access to only one spawning area and a mean of 93% of the individuals sampled was estimated to be alevins (Figure 2).

**FIGURE 2:**
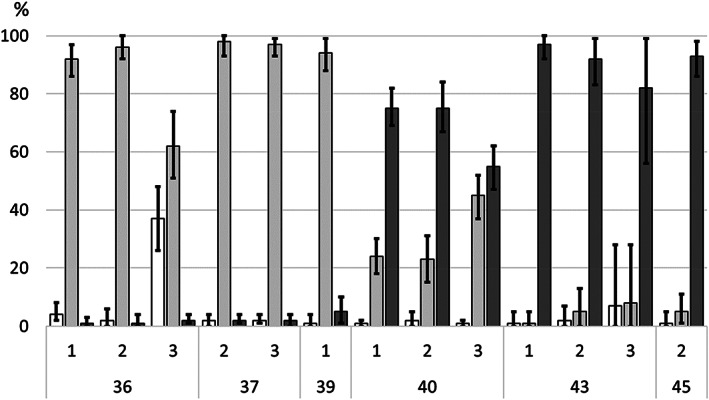
Posterior estimates for proportions of noneyed eggs, eyed eggs and alevins of *Oncorhynchus gorbuscha* redds at three different sampling areas in the River Teno, Garnjarga (1), Kortsam (2) and Badda (3), spanning from early September (week no. 36) to early November (week no. 45). Bars, mean estimates for each proportion; whiskers, 95% probability intervals. (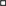) Non‐eyed, (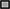) eyed and (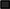) alevins

The number of dead eggs observed was low: the mean numbers across sampling areas varied from 0–3 eggs in September to 0–5 in early October and no dead eggs were observed in late October or November (Table 1).

Water temperature in the Teno mainstem was about 8°C during the first sampling in early September, dropped to 5–6°C in late September, increased to 7–8°C in week 40 and then decreased sharply to 0.1°C and below for the rest of the sampling periods in late October and November (Figure 1).

The exact date of egg deposition and start of incubation for the sampled spawning redds was naturally unknown, so we used our own visual observations of spawning activity in the sampling areas to create three scenarios for possible egg fertilization times. The first active spawning was observed during 2–3 August, followed by very high numbers of spawning fish and redds present at spawning grounds between 13 and 15 August. Finally, few spawners, mostly females, were still active on 10 September. Thus, three cumulative degree‐day curves were fitted starting on 1 August, 15 August and 10 September (Figure 1).

Earlier studies suggest that the degree‐days (°C) needed from *O. gorbuscha* egg fertilization to eyed stage is about 350–400 (Kraus, 1999) and to hatching varies between 530 and 650 (Bailey *et al*., 1980, Hebert *et al*., 1998; Kraus, 1999). Linking the observed developmental stages of eggs and alevins (Figure 2) to the degree‐days in the Teno River in the three spawning time scenarios (Figure 1), it appears that most spawning and egg fertilization must have followed very closely the earliest scenario, where the starting point was set at 1 August: the about 400°‐days was reached at the end of August, which matched well with the observed 67%–99% of eggs being at eyed stage in early September (Figure 2). Similarly, about 600°‐days matched with our sampling in the first days of October (week 40), when most eggs had already hatched (Figure 2). It appears unlikely that a large part of *O. gorbuscha* egg development in our sampling areas had started after mid‐August (Figure 1).

The early spawning of *O. gorbuscha* in the River Teno is in fairly good accordance with that observed in the Russian White Sea basin (Alekseev *et al*., 2019), where spawning commenced when the water temperature was between 8°C and 10°C. However, Alekseev *et al*. (2019) reported that spawning starts in this area between 10 and 15 August, which appears to be slightly later than in the River Teno. Gordeeva *et al*. (2015) suggested a potential selection for early spawning in the pink salmon population established in their new distribution area in the north‐east Atlantic Ocean. Groot & Margolis (1991) summarized spawning temperatures of *O. gorbuscha* across rivers in the original Pacific distribution area. They varied a lot between rivers: from 5 to 19°C in several rivers in the Russian far east, and between 7 and 18°C in rivers in Alaska and British Columbia, although most typical water temperatures at the onset of spawning period were close to 10°C (Groot & Margolis, 1991).

In 2021, the first ever large population of *O. gorbuscha* that spawned in the Teno River (Sandlund *et al*., 2019; Anon, 2021) started its spawning period much earlier than that typically observed for the native Atlantic salmon (*Salmo salar* L.) in the Teno system (Karppinen & Erkinaro, 2009; Orell *et al*., 2011; Mobley *et al*., 2019), suggesting little disturbance for *S. salar* spawning. However, the latest observations of active *O. gorbuscha* in the spawning grounds were made in mid‐September, which overlaps with observations of spawning of the native anadromous species, *S. salar*, and brown trout (*Salmo trutta* L.), especially in tributaries of the River Teno (early to late September; Orell *et al*. 2011, 2017), some of which were also invaded by *O. gorbuscha* in 2021 (Anon, 2021).

The early spawning time of *O. gorbuscha* and subsequent egg development and hatching observed in the Teno River may affect the likelihood of survival and recruitment. Gordeeva *et al*. (2015) suggested that early egg development and resorption of the yolk sack of alevins is associated with difficulties in starting external feeding and related high mortality. The documented early egg development may have the potential to affect the survival of juvenile *O. gorbuscha* and counteract the recent dramatic increase in spawning population in the River Teno and perhaps elsewhere in the Atlantic area. However, the observed spawning time spanning over a month may demonstrate the potential variation in egg development rate in the River Teno, and selection pressures from local environmental conditions may facilitate better survival for offspring of later spawners (*e.g*., Hebert *et al*., 1998). Additional studies on egg and alevin development and related adaptive survival in the new distribution area are needed to understand the role of temporal variability in spawning time in establishment of successful, self‐sustaining spawning populations of *O. gorbuscha* in the Atlantic area.

## AUTHOR CONTRIBUTIONS

J.E. and P.O. conceptualized the study. H.P conducted the data analysis. All authors participated in fieldwork, sampling, and writing and editing the manuscript.

## ETHICS APPROVAL

Specimens used in this study were sampled during investigations conducted under permission from the Centre for Economic Development, Transport and the Environment of Lapland (LAPELY 567/5713–2018) to the Natural Resources Institute Finland (Luke).
